# Serum folic acid: an effective indicator for arteriogenic erectile dysfunction

**DOI:** 10.3389/fendo.2023.1080188

**Published:** 2023-07-24

**Authors:** Xingliang Feng, Yangyang Mei, Pinpeng Xie, Zhaoyu Xing, Xiaogang Wang, Li Cui, Renfang Xu

**Affiliations:** ^1^ Department of Urology, The Third Affiliated Hospital of Soochow University, Changzhou, Jiangsu, China; ^2^ Department of Urology, The First People’s Hospital of Changzhou, Changzhou, Jiangsu, China; ^3^ Department of Urology, Jiangyin People’s Hospital of Jiangsu Province, Jiangyin, China; ^4^ Department of Clinical Medicine, The Second School of Clinical Medicine, Anhui Medical University, Hefei, China; ^5^ Department of Urology, The First Affiliated Hospital of Anhui Medical University, Hefei, Anhui, China

**Keywords:** folic acid, arteriogenic erectile dysfunction, peak systolic velocity, penile color Doppler ultrasonography, effective indicators

## Abstract

**Background:**

The present study is the first to explore the correlation between serum folic acid (FA) level and penile arterial peak systolic velocity (PSV) as measured via penile color Doppler ultrasonography (PDU), which directly reflects endothelial function in the penile artery.

**Materials and methods:**

A total of 244 consecutive erectile dysfunction (ED) patients and 72 healthy controls, recruited from the Andrology department and the Healthy Physical Examination Center of our hospital, respectively, from June 2020 to April 2022, were included in the study. Serum FA was measured in ED patients and healthy controls, and PDU examinations were conducted for all eligible ED patients. The Pearson method was used to evaluate the correlation between FA levels and PDU parameters in ED patients. A receiver operating characteristic (ROC) curve analysis was also performed to calculate the sensitivity and specificity of these parameters for prediction of arteriogenic ED.

**Results:**

After the PDU test, the average serum FA level among patients diagnosed with arteriogenic ED was 8.08 ± 2.64 ng/ml, lower than the average of 10.78 ± 2.87 ng/ml among healthy controls. There were no statistically significant inter-group differences on any basic parameters, including age, body mass index, fasting blood glucose, total cholesterol, and triglyceride. For further analysis, we divided the arteriogenic ED group into three subgroups by PSV range to compare serum FA levels among these subgroups. The mean FA levels in each of these groups were 5.97 ± 1.51ng/ml, and 8.21 ± 2.37ng/ml, and 10.55 ± 2.56ng/ml, while the corresponding PSV values were 15.75 ± 2.39cm/s, 23.53 ± 2.19cm/s, and 32.72 ± 1.64cm/s. Overall, a positive correlation between PSV and FA level was found among patients with arteriogenic ED (r=0.605, P<0.001). Furthermore, when FA level was used, with a cut-off value of 10.045 ng/ml, as a criterion to distinguish patients with arteriogenic ED from healthy controls, the area under the curve (AUC) was 0.772 (95% confidential interval: [0.696, 0.848]), for a sensitivity of 0.611 and specificity of 0.824.

**Conclusion:**

Serum FA level is positively correlated with PSV in ED patients, and has the ability to distinguish patients with arteriogenic ED from healthy controls. Taking these findings together, FA deficiency should be regarded as an independent risk factor for arteriogenic ED.

## 1 Introduction

Erectile dysfunction (ED), defined as the consistent inability (lasting for at least 6 months) to obtain and/or maintain an erection sufficient for sexual intercourse (1), is one of the most common forms of sexual dysfunction in men. The global prevalence and incidence of ED have been growing, and it is predicted that incidence will have increased to 332 million cases by the end of 2025 (2). Although the disorder presents no immediate threat to the lives of patients, it can exert an ongoing effect not only the physical and psychosocial health of patients, but also on their sexual partners’ quality of life (3). Although ED has been found to have multifactorial etiology, including neurogenic, hormonal, psychogenic, and iatrogenic causes (4), vascular causes have been found to account for the majority of ED cases owing to the specific vascular network of the penis (5). It is estimated that 25%-70% of ED cases could be attributed to vascular factors (3); these cases are referred to as vascular ED and include arteriogenic ED, venogenic ED, and mixed ED (6). Currently, the risk factors for vascular ED that are recognized based on clinical investigations include hypertension, diabetes mellitus, and hyperlipidemia, which can lead to endothelial dysfunction of the penile vessels (7). Beyond the destructive effects caused by the vasculature-damaging substances involved in these conditions, folic acid (FA) deficiency may also play a vital role in the pathogenesis of ED by decreasing the function of the penile artery endothelium.

FA, a water-soluble B vitamin, may improve endothelial function by acting on the process of nitric oxide (NO) metabolism (8), which not only plays an essential role in maintaining endothelial function, but also participates in the process of erection via the cyclic guanosine monophosphate (cGMP) pathway (9). FA is indispensable for the structural integrity of endothelial NO synthase (eNOS), which is expressed abundantly in the endothelium of the penile artery. When FA deficiency occurs in a patient, eNOS becomes uncoupled (10), resulted in reduced formation of NO. Naturally, this would be expected to damage endothelial function. Several case–control studies have explored the association between FA level and ED, and these have reached the conclusion that as serum FA level decreases, ED severity increases; these results have been obtained both in studies comparing FA levels between ED patients and healthy controls (11, 12) and in studies comparing FA levels in ED patients with different degrees of ED (13). Furthermore, our group has also conducted a carefully designed meta-analysis to explore the association between serum FA level and ED severity, and to explore the potentially beneficial effects of FA supplementation in ED patients (10). However, a major limitation of all these studies should be pointed out: namely, no studies have related serum FA level directly to endothelial function, considering the vital role of FA in maintaining endothelial function.

Penile color Doppler ultrasonography (PDU) is considered the “gold standard” for diagnosis of vascular ED via measurement of the relevant parameters after artificial induction of penile erection through intra-cavernous injection (ICI) of vasoactive agents (14). Peak systolic velocity (PSV), the most commonly used parameter, can be measured for direct evaluation of the penile arterial supply (15). Additionally, PSV has been found to be closely correlated with probability of future cardiovascular events (16). When PSV is <35cm/s, arterial insufficiency of the penile artery can be diagnosed (17). Additionally, the degree of decline in PSV has been found to be related to the severity of damage to the penile artery, determined mainly by the function of the penile endothelium.

Considering the aforementioned limitations of the existing published studies, the aim of the present prospective study was to further explore the correlation between serum FA level and PSV in the PDU test in arteriogenic ED patients, while also enrolling more participants to compensate for the small sample size of previous studies. To the best of our knowledge, this is the first study to evaluate the effect of FA on male erectile function using PDU parameters. A positive finding would definitively confirm the role of FA in the pathology of vascular erectile dysfunction, and provide a certain amount of evidence for the use of FA in andrology to protect and/or restore erectile function in ED patients.

## 2 Materials and methods

This prospective study was conducted in compliance with the Helsinki Declaration (18) (as revised in 2013). It was approved by the ethics committee of the First Affiliated Hospital of Anhui Medical University and the ethics committee of the First People’s Hospital of Changzhou prior to the start of the study, and all participants signed a statement of informed consent before participating.

### 2.1 Study participants

A total of 285 ED patients who attended the Urology and Andrology department of our hospital from June 2020 to April 2022, all aged between 18 and 60 years and regularly engaging in sexual intercourse, were initially included in our study. The inclusion criteria for ED patients were: (a) heterosexual men older than 18 years engaging in regular sexual intercourse (at least once per week with a fixed sexual partner); (b) diagnosed with ED for at least 6 months based on the International Index of Erectile Function-5 (IIEF-5 <=21); (c) the ability to comprehend and complete the relevant questionnaires and provide informed consent. Potentially eligible participants were excluded if they met any of the exclusion criteria: (a) psychogenic ED or neurogenic ED; (b) systematic diseases such as diabetes mellitus, hypertension, hyperhomocysteinemia, coronary arterial diseases, liver dysfunction, or renal dysfunction; (c) hormonal disorders such as hyperhomocysteinemia or hypogonadism; (d) use of drugs affecting endocrine status, erectile function, or vitamin levels in the body; (e) use of phosphodiesterase-5 inhibitors (PDE5i) within the last 6 months, either chronically or on demand; (f) having undergone penile prothesis operation.

For comparison purposes, 72 healthy control participants were also enrolled in the study; these participants were recruited from the Healthy Physical Examination Center of our hospital during the same time period. They all completed the IIEF-5 to assess their erectile function in the preceding 6 months. All the inclusion and exclusion criteria also applied to the healthy control participants. In addition to the evaluation of erectile function using the IIEF-5, all healthy controls underwent blood tests, including for serum testosterone, fasting sugar, total cholesterol, total triglyceride, and folic acid level under fasting conditions at 8:00 AM. Due to the invasive nature of the PDU test and associated ethical restrictions, PDU tests were not conducted in the healthy controls.

### 2.2 Study design

The patient sample and healthy controls were recruited from the outpatient department and the Healthy Physical Examination Center, respectively, during the same time period. First, a detailed clinical investigation was conducted for each participant (both patients and controls) by a senior andrologist using a pre-designed questionnaire, which included their medical history, sexual history, history of diseases and use of medication, and the IIEF-5. Next, all the ED patients meeting the inclusion criteria proceeded to undergo nocturnal penile tumescence and rigidity (NPTR) tests for identification of psychogenic ED (19); such patients were excluded from this study, and the remaining eligible patients continued with the next phase of detailed examination. In this third phase, patients with organic ED underwent the PDU test for evaluation of penile hemodynamics. Finally, laboratory tests were conducted for all eligible ED patients and healthy controls to measure the serum indicators associated with ED. Due to ethical restrictions, healthy controls did not undergo the PDU test or the NPTR test.

### 2.3 Blood tests

The blood tests conducted for each patient included serum testosterone, fasting sugar, total cholesterol, total triglyceride, and folic acid level. Blood samples were collected from the antecubital vein between 6:00 and 8:00 AM under fasting conditions to avoid influencing the results, especially the measures of fasting sugar, total cholesterol, total triglyceride, and so on. Blood samples were analyzed within 1h after venipuncture. For the sugar and lipid analyses, a Fully Automatic Biochemistry Analyzer (Modular Analytics, Roche8001, USA) was used to perform the analyses. For testosterone and FA, a fully automated chemiluminescence immunoassay system (ADVIP Centaur, Siemens Inc., German) was used.

### 2.3 NPTR test

NPTR tests were administered to ED patients using the RigiScan Monitor device [GOTOP Inc., USA]. The accuracy of the results of the NPTR test is heavily dependent on the patient’s sleep quality. Therefore, the test was conducted for each patient on two consecutive nights, beginning at 21:00 PM and ending at 8:00 AM the next day. The intention of the first overnight test was to enable the patient to adapt to the device; the data from the second overnight test data were recorded for diagnosis using the RigiScan Plus software, version 4.0. The parameters of the NPTR test recorded for analysis included base and tip tumescence and rigidity, number of events, and duration of best episodes. In addition to improving the patients’ adaption to the device, a restful night’s sleep was also required to improve the reliability of the NPTR test. It was critical for patients to avoid napping, caffeine, and alcohol intake during the daytime prior to the examination. Furthermore, patients were advised to empty their bladder and bowel prior to falling asleep, in order avoid disturbance of their sleep during the nighttime, which would have negative effects on the NPTR results.

### 2.4 PDU test

For patients with abnormal NPTR results, a PDU test was administered to further explore the type of ED that was present. To reduce inter-examiner variability, a senior sonographer with 5 years of PDU experience was responsible for all PDU tests. The PDU test was conducted using an AixplorerTM ultrasound system (Supersonic Imagine S.A., Aix-en-Provence, France) with a SuperLinearTMSL15-4 probe (Frequency: 4–15 MHz). Following the standard operating procedures for PDU (20), the sonographer was blinded to the clinical characteristics of the ED patients, in order to avoid subjective influence on the PDU test. Another statement of informed consent was signed by patients before the PDU test; this material indicated the risks of the PDU test, including pain, infection, and priapism, which mainly arise as a result of the intracavernosal injection (ICI) of vasoactive agents. The parameters recorded during the PDU test included peak systolic velocity (PSV), end-diastolic velocity (EDV), and resistive index (RI = PSV–EDV/PSV) of both cavernous arteries. Recording of these parameters began with the penis in the flaccid state and continued for 25 minutes after the ICI, at intervals of 5 minutes. To induce optimal erection, the ICI of 10μg alprostadil was administered close to the base of the patient’s penis, with simultaneous provision of audio-visual sexual stimulation (AVSS).

### 2.5 Outcome measures

The IIEF-5 was used to evaluate erectile function in all participants. A diagnosis of ED was confirmed if the total score on the IIEF-5 was less than 22. In addition to the IIEF-5, the participant’s age and BMI were also collected for further analyses. The biochemical parameters measured via the blood tests performed for each patient included fasting blood glucose (FBG), total cholesterol (TC), triglyceride (TG), total testosterone, and folic acid. For the NPTR test, an effective erectile event was defined as a penile erection lasting for ten minutes or more with at least 60% rigidity recorded at the tip of the penis (21). For the PDU test, the highest PSV and lowest EDV were recorded. Cavernous artery flow was considered normal if the PVS was >35cm/s, and corporeal veno-occlusive function was considered normal if the EDV was <5cm/s and the RI was >0.9. Following the reference values for the PDU test, arterial insufficiency was diagnosed when PSV was below 35cm/s for both cavernous arteries and the EDV was also below 5cm/s; corporeal veno-occlusive dysfunction was diagnosed when EDV was above 5cm/s and RI was below 0.75, with normal PSV. When all these parameters were abnormal, mixed vascular insufficiency was diagnosed. In contrast, non-vascular ED was diagnosed when all these parameters were normal.

### 2.6 Statistical analyses

All statistical analyses were performed using SPSS software version 16.0 (SPSS, Inc., Chicago, IL, USA). Differences were considered statistically significant if the corresponding P value was less than 0.05, and all statistical comparisons were two-sided. Data are presented in the form of means with the corresponding standard deviation (SD) in cases where the variable follows the normal distribution, as indicated by the Kolmogorov–Smirnov test. Otherwise, data are presented in the form of median (first quartile−third quartile). For comparisons, a one-way analysis of variance (ANOVA) was conducted in cases where a continuous variable met the assumptions of normal distribution and homogeneity; otherwise, the Kruskal–Wallis H test was employed. Multiple comparisons among different groups were also carried out as needed using appropriate methods. The Pearson method was used to evaluate correlation coefficients. Finally, receiver operating characteristic (ROC) curve analysis was performed to calculate the sensitivity and specificity of various relevant parameters for prediction of arteriogenic ED.

## 3 Results

**Figure 1:**
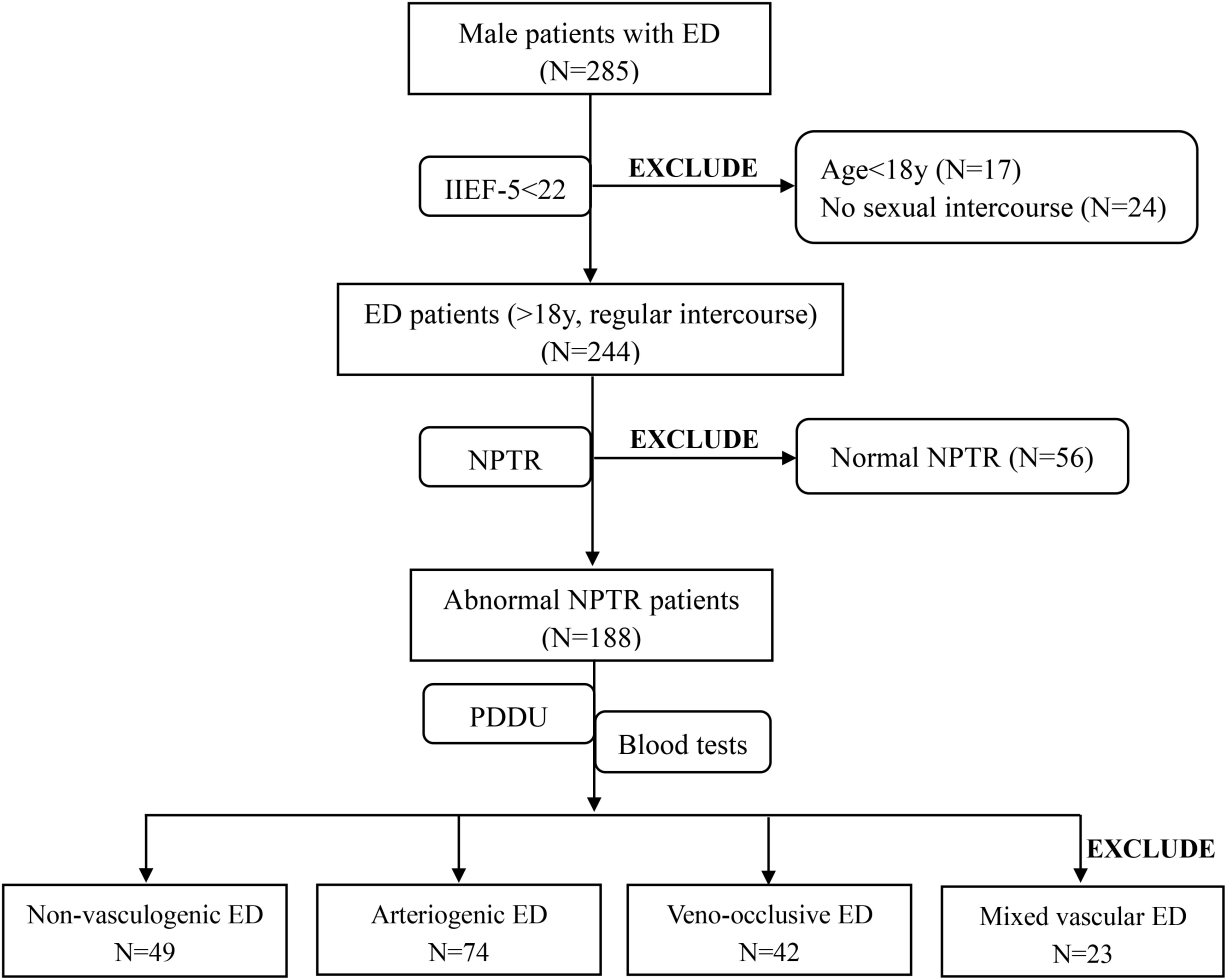
Flow diagram illustrating selection of the study population.

Initially, a total of 285 ED patients from the Urology and Andrology department were screened for participation. 41 ED patients were excluded from our study, 17 of whom were younger than 18s, and 24 of whom were not engaging in regular sexual intercourse. Ultimately, 244 consecutive eligible ED patients and 72 healthy controls were included in the present study. After completion of the NPTR test, we excluded a further 56 patients due to normal NPTR results. The PDU test was conducted with the remained patients to further explore the origin of their ED. Based on the PDU test, 23 additional ED patients who were diagnosed with mixed vascular ED with arterial insufficiency and corporeal veno-occlusive dysfunction were eliminated from inclusion in further analyses. Thus, the final dataset consisted of data from 74 patients diagnosed with arteriogenic ED, 42 patients diagnosed with veno-occlusive ED, and 49 patients diagnosed with non-vasculogenic ED. A further 72 healthy subjects were also enrolled in our study for comparison. The details of the screening process for ED patients are shown in **Figure 1** in the form of a flow diagram.

The demographic and clinical characteristics of the study population are shown in **Table 1**, summarized across four groups (the control group, the non-vascular ED group, the veno-occlusive ED group, and the arteriogenic ED group). There were no statistically significant differences among the groups on any of the basic parameters, including age, BMI, FBG, TC, and TC. In terms of IIEF-5 score, as an assessment of erectile function, the mean scores were 23.94 ± 1.09, 12.35 ± 4.70, 11.71 ± 3.57, and 12.49 ± 4.48 for the control group, the non-vascular ED group, the veno-occlusive ED group, and the arteriogenic ED group, respectively (P<0.001). In a further comparison among the latter three groups only, there were no statistically differences in IIEF-5 score (P>0.05). For serum FA level, the mean values were 10.78 ± 2.87 ng/ml, 10.93 ± 3.57 ng/ml, 10.24 ± 2.41 ng/ml, and 8.08 ± 2.64 ng/ml for each of the four groups (with significant differences indicated, P<0.001).

**Table 1:** Demographic data and clinical parameters of study participants.

	Control (N=72)	Non-vasculogenic ED (N=49)	Veno-occlusive ED (N=42)	Arteriogenic ED (N=74)	P
Age, years	35.06 ± 10.60	34.96 ± 8.72	36.64 ± 8.94	37.11 ± 9.55	0.489**^a^**
BMI, kg/m^2^	24.76 ± 1.20	24.38 ± 2.26	23.84 ± 1.80	24.70 ± 2.28	0.111**^a^**
FBG, mmol/L	4.88 ± 0.69	4.97 ± 0.72	4.94 ± 0.50	4.72 ± 0.65	0.139**^a^**
TC, mmol/L	4.46 ± 1.23	4.40 ± 1.21	4.54 ± 1.00	4.45 ± 1.27	0.955**^a^**
TG, mmol/L	1.77 ± 0.71	1.78 ± 0.77	1.57 ± 0.67	1.58 ± 0.66	0.211**^a^**
Total T, nmol/L	20.82 ± 5.34	20.82 ± 4.90	20.20 ± 3.86	20.87 ± 3.95	0.877**^a^**
FA, ng/ml	10.78 ± 2.87	10.93 ± 3.57	10.24 ± 2.41	8.08 ± 2.64**^c^**	**<0.001^a^**
IIEF-5 score	23.94 ± 1.09	12.35 ± 4.70	11.71 ± 3.57	12.49 ± 4.48**^d^**	**<0.001^b^**
PDU parameters					
PSV, cm/s	—	48.18 ± 9.91	46.47 ± 7.61	23.29 ± 9.20	**<0.001^a^**
EDV, cm/s	—	-1.69 ± 2.97	7.73 ± 1.64	0.00 ± 1.71	**<0.001^a^**

ED, erectile dysfunction; BMI, body mass index; FBG, fasting blood glucose; TC, total cholesterol; TG, triglyceride; Total T, total testosterone; FA, folic acid; IIEF-5, International Index of Erectile Function-5; PDU, penile Doppler ultrasonography; PSV, peak systolic velocity; EDV, end-diastolic velocity; RI, resistive index.

**^a^** statistically significant difference as indicated by one-way ANOVA; **^b^** statistically significant difference as indicated by Kruskal–Wallis H test; **^c^** statistically significant difference between the arteriogenic ED group and the other three groups; **^d^** no statistically significant difference among the non-vasculogenic, veno-occlusive ED, and arteriogenic ED groups.

The bold values means the calculated p values <0.001.

In order to further explore the association between PSV and serum FA level, we divided the arteriogenic ED group into three subgroups according to PSV: PSV<20cm/s, PSV in the range from 20cm/s to 30cm/s, and PSV in the range from 30cm/s to 35cm/s. As shown in **Table 2**, no significant differences were found among these three groups in terms of any of the basic parameters, including age, BMI, FBG, TC, and TC. However, statistically significant differences among these groups were observed for all of the remaining parameters, including IIEF-5, PSV, and FA, with P <0.001 in all cases. For FA, the mean levels were 5.97 ± 1.51ng/ml, 8.21 ± 2.37ng/ml, and 10.55 ± 2.56ng/ml; for PSV, the mean values were 15.75 ± 2.39cm/s, 23.53 ± 2.19cm/s, and 32.72 ± 1.64cm/s.

**Table 2:** Folic acid levels in patients with arteriogenic ED, grouped by PSV range.

	Arteriogenic ED (N=74)			P
	PSV <20cm/s (N=20)	PSV 20–30cm/s (N=39)	PSV 30–35cm/s (N=15)	
Age, years	37.15 ± 11.74	36.18 ± 8.20	39.47 ± 9.86	0.532**^a^**
BMI, kg/m^2^	25.24 ± 2.37	24.59 ± 2.30	24.29 ± 2.12	0.433**^a^**
FBG, mmol/L	4.53 ± 0.59	4.85 ± 0.62	4.64 ± 0.77	0.193**^a^**
TC, mmol/L	4.53 ± 1.52	4.41 ± 1.17	4.29 ± 1.22	0.722**^a^**
TG, mmol/L	1.73 ± 0.75	1.55 ± 0.60	1.46 ± 0.71	0.466**^a^**
Total T, nmol/L	20.63 ± 4.35	20.70 ± 3.87	21.64 ± 3.78	0.702**^a^**
FA, ng/ml	5.97 ± 1.51	8.21 ± 2.37	10.55 ± 2.56	**<0.001^a^**
IIEF-5 score	7.5 (5.25-9.75)	12 (10-14)	18 (17-20)	**<0.001^b^**
PSV, cm/s	15.75 ± 2.39	23.53 ± 2.19	32.72 ± 1.64	**<0.001^a^**

ED, erectile dysfunction; BMI, body mass index; FBG, fasting blood glucose; TC, total cholesterol; TG, triglyceride; Total T, total testosterone; FA, folic acid; IIEF-5, International Index of Erectile Function-5; PDU, penile Doppler ultrasonography; PSV, peak systolic velocity; EDV, end-diastolic velocity; RI, resistive index.

**^a^** statistically significant difference as indicated by one-way ANOVA; **^b^** statistically significant difference as indicated by Kruskal–Wallis H test. The bold values means that there were significant difference between the three group on the compared characteristics.

**Figure 2:**
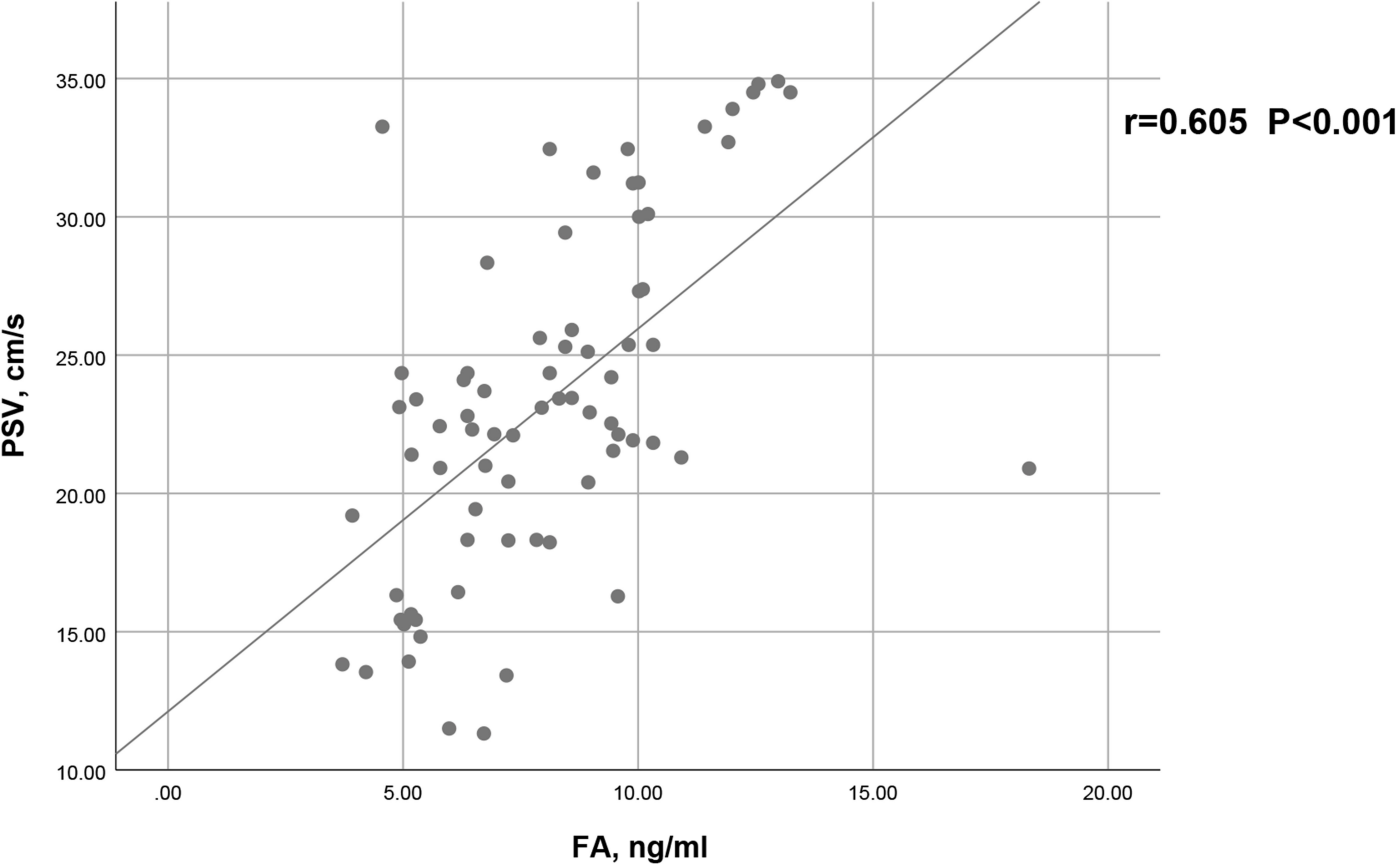
Correlation between serum FA level and PSV in patients with arteriogenic ED (r=0.605, p<0.001).

**Figure 3:**
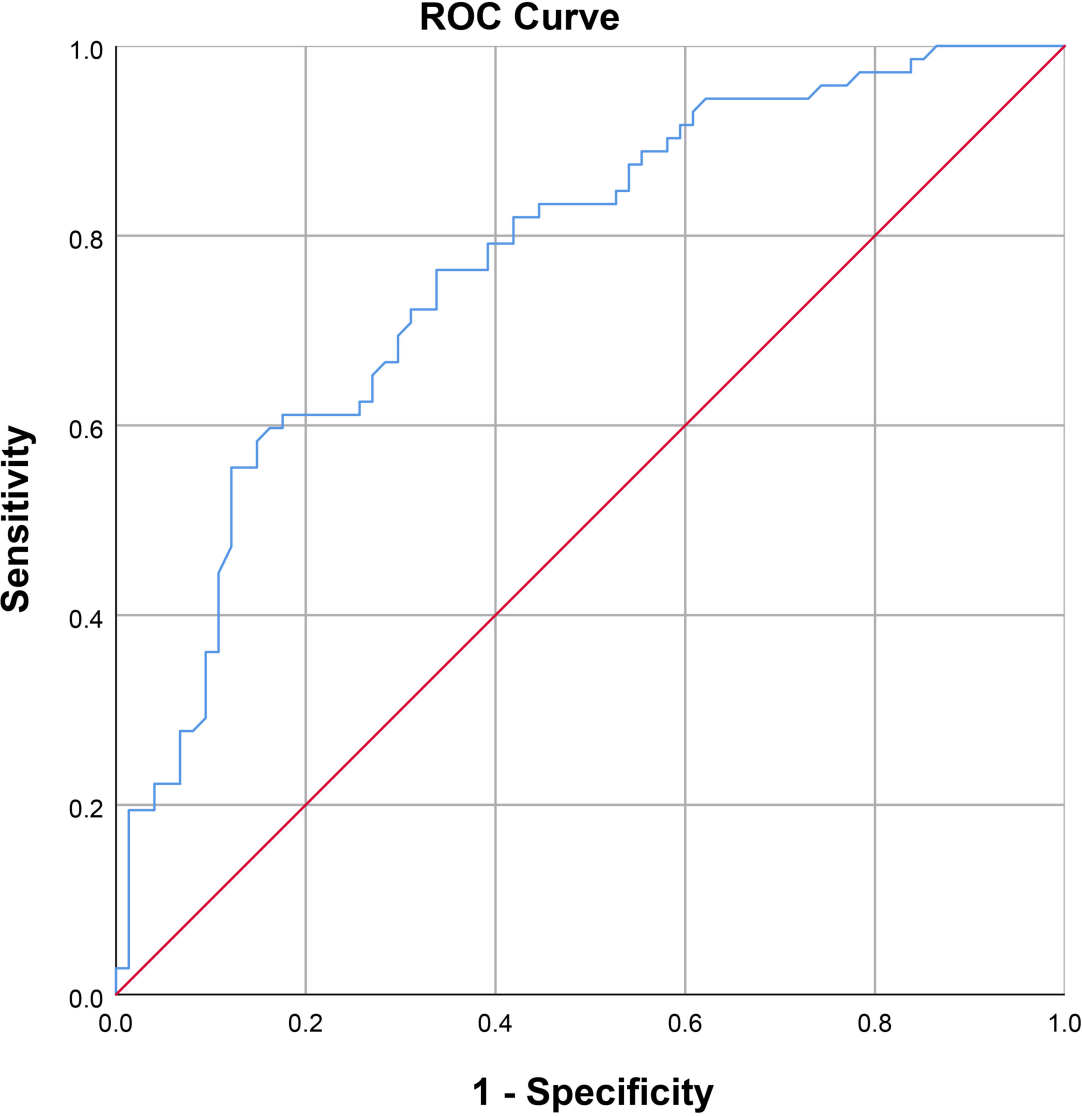
Receiver operating characteristic curve for serum FA level for distinguishing patients with arteriogenic erectile dysfunction (ED) from healthy subjects (AUC=0.772 [0.696-0.847]).

As shown in **Figure 2**, a positive correlation between PSV and FA level was observed in arteriogenic ED (r=0.605, P<0.001). To assess the value of FA level in distinguishing patients with arteriogenic ED from healthy controls, a ROC curve analysis was performed; the area under the curve (AUC) was 0.772 with the 95% confidence interval [0.696, 0.848]. The optimal cut-off value of FA for prediction of arteriogenic ED was 10.045 ng/ml, with sensitivity of 0.611 and specificity of 0.824. These results are shown in **Figure 3**.

## 4 Discussion

Prior to our study, a growing number of case–control studies had been conducted to verify the role of FA in the etiology of ED by comparing FA levels between ED patients and healthy controls (11, 13, 22, 23). Our results further confirmed that serum FA levels are meaningfully lower in patients with arteriogenic ED than in healthy controls (8.08 ± 2.64ng/ml vs. 10.78 ± 2.87ng/ml, P<0.001). More importantly, serum FA level has a positive influence on PSV in the PDU test (r=0.605, P<0.001). Consequently, we can conclude that serum FA level is important for the function of the endothelium, and also for erectile function. Our results demonstrated that FA deficiency should be considered an independent risk factor for arteriogenic ED.

Cardiovascular disease (CVD) has now become the leading cause of morbidity and mortality worldwide (24). Interestingly, ED has been considered to be an independent risk factor for CVD, and even a sentinel symptom of subclinical CVD (25). The two conditions share a number of risk factors, including glucose metabolism, hypertension, dyslipidemia, and obesity, among others (26). Common underlying mechanisms linking the two have also been identified, such as inflammation (27), atherosclerosis (28), and endothelial dysfunction (29). Impairment of endothelial function as a result of these risk factors reduces the capacity for vascular smooth muscle relaxation. When these risk factors are present in men, an interesting phenomenon has often been found to emerge clinically, in which ED precedes symptoms of CVD by 2–3 years (30). The artery size hypothesis has been widely accepted as an explanation of the phenomenon in which ED develops before CVD (31). First, arteries of different diameters show different degrees of susceptibility to systemic risk factors, meaning that the smaller penile artery may be more susceptible than larger cardiovascular arteries (32). Second, the same degree of artery damage compromises blood flow more profoundly through the smaller arteries of the penis than through the larger vessels of the heart and limbs (33).

NO plays an important role in the initiation and maintenance of the erectile process through the cyclic guanosine monophosphate (cGMP) pathway. In order to produce NO, it is necessary to maintain the structural integrity of eNOS, and FA plays a vital role in promoting the morphological integrity of eNOS via tetrahydrobiopterin (BH4), an essential cofactor of eNOS (34). BH4 works to promote the integrity of eNOS by keeping the oxidase and reductase subunits linked together, and also by increasing the affinity of the other cofactors for eNOS (35). Consequently, FA deficiency is responsible for BH4 deficiency, which further responsible for the uncoupling of eNOS. The uncoupling of eNOS represents a close link between FA deficiency and endothelial dysfunction.

The first study to explore the correlation between serum FA level and ED was conducted by Yan et al., in which FA levels in ED patients (n=42) and healthy controls (n=30) were compared (12). In their study, serum FA levels in ED patients were found to be 7.61 ± 3.97ng/ml, lower than the 12.23 ± 5.76ng/ml observed in healthy controls. The erectile function of ED patients was assessed only via IIEF-5 scores. The authors additionally explored the correlation between FA level and IIEF-5; the results indicated a positive correlation between the two (r=0.589, P<0.001), smaller than that observed in our study (r=0.605, P<0.001). PSV was measured in our study for more direct evaluation of endothelial function. Therefore, our findings verify the vital role of FA in the pathogenesis of arteriogenic ED.

A randomized double-blind clinical trial was conducted in 2013 to assess the efficacy of combination therapy with FA and tadalafil for ED (36). The researchers found that this combination therapy could improve IIEF scores to a greater extent than tadalafil monotherapy (5.14 ± 3.84 vs. 1.68 ± 0.99, P<0.001). Tadalafil, a phosphodiesterase type 5 inhibitor (PDE5I), has been used as a first-line therapy for ED, as have other such drugs such as sildenafil and avanafil (37). The main mechanism of action of tadalafil is the inhibition of cGMP catabolism. Meanwhile, NO functions as a master regulator in production of cGMP by activating soluble guanylyl cyclase (38). Therefore, the absence of NO in the penile vasculature should reduce the therapeutic efficacy of tadalafil.

Another explanation for the correlation between FA and ED is the metabolism of cysteine. Cysteine is a sulfur-containing amino acid synthesized from methionine, which is among the essential amino acids for the human body (39). Cysteine is also an important regular of eNOS (40), inhibiting eNOS activity via the protein kinase C and threonine 495 phosphorylation pathways (41). FA is an essential cofactor of this enzyme, which is responsible for the metabolism of cysteine by remethylating it to methionine (12). Therefore, some scholars have concluded that the underlying mechanism of ED in cases of FA deficiency is associated with the metabolism of cysteine, leading to hyperhomocysteinemia in the plasma. Researchers have found that serum FA level is negatively correlated with serum cysteine level in ED patients (r=-0.508, P<0.01). Furthermore, FA supplementation has been found to decrease serum cysteine levels (0.19umol/l vs. 2.84umol/l, P<0.001), accompanied by the simultaneous improvement of erectile function in ED patients (15.5 vs 6.4, P<0.001). However, in another study conducted to explore the correlation between serum FA levels and serum cysteine concentration (11), no correlation was found between the two (r=-0.613, P=0.372), demonstrating that FA may exert a direct effect on endothelial function, beyond the pathway involving metabolism of cysteine. A carefully designed meta-analysis conducted by our group also verified this conclusion by combining the results of observational and interventional studies with larger sample sizes (10). Evidently, this conclusion is also consistent with the present results, in which serum FA level was found to be closely correlated with endothelial function, as measured by the PDU examination.

Several limitations should be borne in mind in the clinical interpretation of our conclusions. First, the lack of an intervention to raise FA levels means that this finding requires further confirmation in well-designed studies, ideally in randomized controlled trials. Second, we failed to measure serum cysteine levels, instead simply excluding patients with hyper-cysteine. Third, we did not report the correlation between serum FA level and future cardiovascular disease in our ED patients, all of whom are currently under follow-up by our group. Despite these limitations, the strengths of our study make it worthwhile. First, the enrollment of a larger sample than earlier studies makes our conclusion more convincing. Second, this study is the first to have demonstrated an association of serum FA level with PSV, this being a more direct and straightforward reflection of endothelial function. In the future, the interventional studies should be conducted with a larger sample size and with an appropriate study population to demonstrate the positive role of FA in treatment of vascular ED, especially arteriogenic ED. Certainly, basic animal studies should be performed to explore the pathogenesis of ED arising from FA deficiency.

## 5 Conclusions

In conclusion, a significant association between PSV of the cavernous artery and serum FA level was found in patients with arteriogenic ED. Specifically, PSV decreases significantly as serum FA level decreases, and FA deficiency should be regarded as an independent risk factor for arteriogenic ED. It is critical to perform a large-scale cohort study in the future in order to explore the beneficial effect of FA supplementation for erectile function in patients with arteriogenic ED.

## Data availability statement

The raw data supporting the conclusions of this article will be made available by the authors, without undue reservation.

## Ethics statement

The studies involving human participants were reviewed and approved by the ethics committee of the First Affiliated Hospital of Anhui Medical University (anyiyifuyuanlunshen-kuai-PJ-2019-09-11). The patients/participants provided their written informed consent to participate in this study.

## Author contributions

XF, YM, PX, ZX, and RX conceptualized and designed the study and are responsible for the work in its entirety. XF, PX, and YM conducted the data acquisition. XF, PX, YM, XW, and LC carried out the analysis of the data. All authors participated in drafting of the manuscript and critical revision of the article. All authors contributed to the article and approved the submitted version.
