# Supplementary material for: Serum folic acid: an effective indicator for arteriogenic erectile dysfunction
Source: Front Endocrinol (Lausanne). 2023 Jul 24;14:1080188. doi: 10.3389/fendo.2023.1080188 (PMC10405823; doi:10.3389/fendo.2023.1080188)
Supplement: Supplementary file 1 [file DataSheet_1.docx]

**The 5-item version of the International Index of Erectile Function (IIEF-5) questionnaire**

**Over the past six months:**

**Q1.** How do you rate **your confidence** that you could get and keep an erection?

A. Very low (1)

B. Low (2)

C. Moderate (3)

D. High (4)

E. Very high (5)

**Q2.** When you had erections with sexual stimulation, **how often** were your erections hard enough for penetration?

A. Almost never/never (1)

B. A few times (much less than half the time) (2)

C. Sometimes (about half the time) (3)

D. Most times (much more than half the time) (4)

E. Almost always/always (5)

**Q3.** During sexual intercourse, **how often** were you able to maintain your erection after you had penetrated (entered) your partner?

A. Almost never/never (1)

B. A few times (much less than half the time) (2)

C. Sometimes (about half the time) (3)

D. Most times (much more than half the time) (4)

E. Almost always/always (5)

**Q4.** During sexual intercourse, how difficult was it to maintain your erection to completion of intercourse?

A. Extremely difficult (1)

B. Very difficult (2)

C. Difficult (3)

D. Slight difficult (4)

E. Not difficult (5)

**Q5.** When you attempted sexual intercourse, **how often** was it satisfactory for you?

A. Almost never/never (1)

B. A few times (much less than half the time) (2)

C. Sometimes (about half the time) (3)

D. Most times (much more than half the time) (4)

E. Almost always/always (5)

The IIEF-5 score is the sum of the ordinal responses to the five items; thus, the score can range from 5 to 25.

No ED: 22-25; Mild ED: 12-21; Moderate ED: 8-11; Severe ED: <7.
